# Yellow Fever—New Theory of Its Propagation

**Published:** 1878-09-20

**Authors:** 


### EDITORIAL AND MISCELLANEOUS

☞All communications relating to the business of THE RECORD for the years 1877 and 1878, must be addressed DR. R. C. WORD, Managing Editor Southern Medical Record, Atlanta, Ga.

☞Brief and practical communications are solicited on all subjects pertaining to medicine; also reports o [illegible]ses in practice.

☞Send money by check, postal order or registered letter.

☞Write your name, post-office, county and State plainly.

### YELLOW FEVER—NEW THEORY OF ITS PROPAGATION.

The existence of a wide-spread epidemic of yellow fever in the Mississippi valley affords an opportunity of expressing our views as to its origin and propagation.

Yellow fever prevails under well recognized atmospheric and telluric influences—conditions in many respects similar to those attending the prevalence of malarial fevers. For a number of years the writer has believed that the yellow fever poison resides in the spores (seeds) of a plant which, when introduced into the blood of the human being, produce a virulent blood poisoning, inducing those changes in the system, called yellow fever. The yellow fever plant, according to our view, is not identical with the malarial plant. It is as distinct from the yellow fever plant as it is from the typhoid fever fungus. They are all microscopical plants: and while the malarial plant has never been satisfactorially isolated, it is believed the yellow fever fungus has been discovered by Dr. LeHardy, of Savannah, Ga. The typhoid fever fungus has also been detected, and the dejections of typhoid fever patients have been seen to contain myriads of spores—confirmatory of the theory of fungus poisoning as the true cause of typhoid fever. These plants grow, develop and fructify under specific influences. The malarial and yellow fever plants seem to possess low vitalities. They are easily destroyed by cold. The spores of the malarial plant seem to have greater resisting power to cold than those of the yellow fever fungus. The spores of the typhoid fever plant—as well as the plant itself—seem to have very great powers of resistance to cold. It is largely a winter plant.

The conditions for the full development of the yellow fever plant seem to be these:

A mild winter preceding an epidemic.

Excessive rain falls, saturating the soil.

Large arears of stagnant water.

Contigousness to the sea or rivers.

Continuous warm weather—the thermometer at or above 80°F.

Limited altitude above the sea level.

It will be seen that the conditions are, in many respects, identical with those attending the production of malaria. There are, however, three points of difference. The full development of the malarial plant is not conditioned upon:

A preceding mild winter.

A themometrical continuous heat at or above 80° F.

A limitation as to altitude.

The yellow fever plant is a product of the tropics. The spores are transmissible. They can be conveyed from point to point in vessels, clothing, goods—probably upon tropical fruits. and in mail matter. The disease produced by this plant is not contagious, and personal contact with the sick is only dangerous to the well because the former undoubtedly become carriers of the spores. The plant is not indiginous, but is a tropical exotic. It is only under the proper conditions it propagates itself in the South. Under these conditions its self multiplication is wonderfully rapid. It may or may not have located itself permanently in our Southern land. Apart from the fungus theory of the origin of yellow fever, many observers assert that the poison must be newly imported before an epidemic can occur. Others deny this. They believe the facts point to its origin in malaria, the poison being intensified. Without attempting to decide the question of fresh annual importations as a condition precedent to every epidemic, the facts bear out the statement that the poison is not identical with that of malaria. It is similar, and in this similarity lies the perplexity. As already shown, the conditions for the propagation of the plants are not identical. The preceding mild winter is not necessary for malarial production. Continuous heat above or at 80°F. is not necessary. Neither does altitude limit the full devel opment of the malarial plant. Yellow fever has never prevailed epidemically, say, 500 to 600 feet, above the ocean. Malarial fevers, of all types, prevail at much higher elevations. The writer has seen pernicious malarial fever—malignant and deadly—at 800 feet above the sea.

Malaria may exist one or more thousands of feet above the ocean, should the local condition become operative. Not so yellow fever. All the conditions—save that of altitude—may be in force and the fever will not prevail. To illustrate: Cincinnati can claim no sanitary superiority over Memphis or Vicksburg. Malaria is common to all portions of the Ohio and Mississippi Vallies, showing the conditions for its production to exist. Nevertheless Cincinnati has so far escaped yellow fever poisoning because, if our theory be correct, it is 500 feet above the sea. It is on the border line. The same may be said of other cities of the Ohio and Mississippi Vallies. Why this exemption? New York and Philadelphia both on higher parallels of latitude, but at lower elevations above the sea, have had epidemics of yellow fever. Our theory gives the explanation. It contradicts no facts. The malarial plant—speaking theoretically—germinates, grows, develops and fructifies at high elevations. The yellow fever plant, a product of the tropics, may germinate, may grow, but will not—according to our theory—fructify (is seedless) at greater elevations than 500 to 600 feet above the sea. In the spores reside its poisoning power. These spores must be scattered, be planted in suitable soil and develop through all the phases to perfect fructification, before an epidemic can prevail.

The malarial plant differs in other peculiarities than those mentioned, from the yellow fever fungus. Malarial spores are not capable of transportation. There is not a case of malarial fever on record that ever originated by spores transported. Malarial fever germs, as ordinarially understood, cannot be conveyed from place to place. It appears to differ from typhoid fever and cholera germs. These are transmissible. Why the spores of the malarial plant seem to be incapable of being transported from point to point may be more apparent than real. It may be due to its universal distribution over the earth, and because it reaches the phase of fructification at all elevations above the sea, where the conditions for its growth may exist. Malarial spores may be floating, like those of the mould fungus, everywhere. It is also probable that, like other fungi, the malarial spores are never all destroyed by cold, but remain dormant until the warmth of early spring, under favorable conditions, awaken them into activity. Yellow fever differs from malarial fevers in another respect. Yellow fever rarely repeats itself in the same person, whereas malarial fever may assault the same person year after year during life.

The yellow fever fungus, being a tropical plant, requires a higher temperature in order that it may complete all its phase of development. It does not—according to the best authorities—reach the phase of fructification only after long continued heat at or above 80° F. Its resistence to cold is feeble. It dies, like the stalk of the banana plant, upon the appearance of the first frost.

Many good observers believe that the yellow fever poison has found a permanent home in the semi-tropical portions of the Union. It will be remembered that New Orleans was, for 80 years, exempt from the fever, when during this time, it had scourged the towns of the sea-board just below. It was not known at Savannah until the year 1807. The conditions for the growth and development of the plant were not operative. Yellow fever patients dying with black vomit, have been repeatedly treated at New Orleans and Savannah without communicating the disease to others. The conditions for the fructification of the plant were absent.

A mild winter precedes an epidemic. During such a winter, along with tropical fruits, in clothing or goods, the spores of the plant may be imported. The moderate temperature does not destroy them. Upon the happening of the necessary conditions—the mercury of the thermometer for long periods remaining at or over 80° F.—the growing plant reaches the phase of fructification, and an epidemic, spreading from the myriads of growing plants and floating spores of the full developed plant, occurs.

If the plant has found permanent lodgement on our Southern sea coast, according to our theory—the spores, year after year, must escape destruction at low temperatures. This is improbable, because the plant does not reach the phase of fructification every year, and the preceding mild winter before an epidemic, could not bestow vitality upon the spores destroyed by the cold of previous severe winters.

It is probable, therefore, that the tropical yellow fever plant upon the approach of cold weather dies, after each epidemic, and that fresh importations become necessary before other epidemics can prevail.

